# A Begomovirus Nuclear Shuttle Protein-Interacting Immune Hub: Hijacking Host Transport Activities and Suppressing Incompatible Functions

**DOI:** 10.3389/fpls.2020.00398

**Published:** 2020-04-08

**Authors:** Laura G. C. Martins, Gabriel A. S. Raimundo, Nathalia G. A. Ribeiro, Jose Cleydson F. Silva, Nívea C. Euclydes, Virgilio A. P. Loriato, Christiane E. M. Duarte, Elizabeth P. B. Fontes

**Affiliations:** Department of Biochemistry and Molecular Biology, National Institute of Science and Technology in Plant-Pest Interactions, Bioagro, Universidade Federal de Viçosa, Viçosa, Brazil

**Keywords:** nuclear shuttle protein, begomoviruses, immune hub, NSP, NSP-interacting proteins, NIG, NSI, NIK

## Abstract

Begomoviruses (*Geminiviridae* family) represent a severe constraint to agriculture worldwide. As ssDNA viruses that replicate in the nuclei of infected cells, the nascent viral DNA has to move to the cytoplasm and then to the adjacent cell to cause disease. The begomovirus nuclear shuttle protein (NSP) assists the intracellular transport of viral DNA from the nucleus to the cytoplasm and cooperates with the movement protein (MP) for the cell-to-cell translocation of viral DNA to uninfected cells. As a facilitator of intra- and intercellular transport of viral DNA, NSP is predicted to associate with host proteins from the nuclear export machinery, the intracytoplasmic active transport system, and the cell-to-cell transport complex. Furthermore, NSP functions as a virulence factor that suppresses antiviral immunity against begomoviruses. In this review, we focus on the protein-protein network that converges on NSP with a high degree of centrality and forms an immune hub against begomoviruses. We also describe the compatible host functions hijacked by NSP to promote the nucleocytoplasmic and intracytoplasmic movement of viral DNA. Finally, we discuss the NSP virulence function as a suppressor of the recently described NSP-interacting kinase 1 (NIK1)-mediated antiviral immunity. Understanding the NSP-host protein-protein interaction (PPI) network will probably pave the way for strategies to generate more durable resistance against begomoviruses.

## Introduction

*Begomovirus* represents the largest genus of the *Geminiviridae* family and consists of whitefly-transmitted single-stranded DNA viruses, which severely inflict several important crops and vegetables in tropical and subtropical regions (Rojas et al., 2018). Species of the *Begomovirus* genus can be either monopartite (with one single genomic component) or bipartite (two genomic components, referred to as DNA-A and DNA-B) (Zerbini et al., 2017; Kumar, 2019). The nuclear shuttle protein (NSP) is encoded by the DNA-B of bipartite begomoviruses, which also encodes the movement protein (MP). MP and NSP are required for systemic infection and act cooperatively to mediate the intra- and intercellular trafficking of viral DNA (Lazarowitz and Beachy, 1999; Gafni and Epel, 2002; Sanderfoot et al., 1996). Pioneering studies with the movement proteins from *Bean dwarf mosaic virus* (BDMV) and *Squash leaf curl virus* (SLCV) have established that NSP facilitates the nucleocytoplasmic translocation of viral DNA via nuclear pores, whereas MP is predominantly involved in mediating the cell-to-cell movement of viral DNA via plasmodesmata (Noueiry et al., 1994; Sanderfoot and Lazarowitz, 1995, 1996).

The host range of begomovirus species depends on multiple molecular interactions involved in both co-opting the cellular machinery and evading the innate immune recognition (for reviews see Hanley-Bowdoin et al., 2013; Li et al., 2018; Kumar, 2019). Failure of any one of these steps can decrease or impair the virus proliferation on a given host. NSP is implicated in both pro- and antiviral interactions, which determine host susceptibility to begomovirus. Based on characterized NSP-interacting proteins, we describe here an NSP-interacting immune hub, as one of the influential spreaders of information from the host immune system and begomovirus virulence strategies. As the combinatorial effects of pro- and antiviral interactions may establish the host susceptibility to a virus, the elucidation of the NSP-host protein-protein interaction (PPI) network is expected to shed light on the mechanisms for engineering resistance to begomoviruses.

## NSP-Interacting Proteins Displaying Proviral Functions

As begomoviruses replicate in the nuclei of infected cells, the nascent viral DNA (vDNA) must be translocated from the nucleus to the cytosol via nuclear pores, throughout the cytoplasm to the cell periphery and then to the adjacent, uninfected cells via plasmodesmata. To promote this typical cell-to-cell movement, like any other plant virus, the DNA-B of begomoviruses encodes a movement protein that increases the size exclusion limit of the plasmodesmata and mediates the viral nucleic acid translocation into adjacent cells (Noueiry et al., 1994). However, the mechanisms of viral DNA exit from the nucleus and its intracytoplasmic movement to the cell periphery are far less understood. It has been conceptually accepted that NSP facilitates the nuclear exit of newly replicated vDNA via nuclear pores, but interactions of the viral protein with the host nuclear transport machinery have not been reported. Consistent with the NSP-mediated nucleocytoplasmic transport of ss-vDNA, NSPs from BDMV and *Abutilon mosaic virus* (AbMV) interact with ss-vDNA and ds-vDNA presumably in the nuclei of infected cells, whereas NSP from SLCV binds only ss-vDNA *in vitro* (Pascal et al., 1994; Rojas et al., 1998; Hehnle et al., 2004). Immunogold labeling of systemically infected leaves localized SLCV NSP in the nuclei of phloem cells (Pascal et al., 1994) and expression of an epitope-tagged AbMV NSP in *Nicotiana benthamiana* cells confirmed its nuclear localization (Kleinow et al., 2009). In transient assays, NSP from different begomoviruses has been shown to be located in nuclei of cells exclusively expressing NSP fusions but is redirected to the cell periphery in the presence of co-expressed MP (Sanderfoot and Lazarowitz, 1995; Sanderfoot et al., 1996; Lazarowitz and Beachy, 1999; Zhang et al., 2001). The identification of an NSP-interacting GTPase (NIG), which interacts with NSP at the cytosolic side of the nuclear pore complex and shares transport properties with the human Rev-Interacting Protein (hRIP), has shed light on the mechanism for the release of NSP-vDNA complex from the nuclear periphery into the cytoplasm (Carvalho et al., 2008a, b). NSP also interacts with MP in the cytoplasm (Carvalho et al., 2008a), which may promote the directionality of virus translocation to the cell surface (Noueiry et al., 1994; Sanderfoot and Lazarowitz, 1995; Frischmuth et al., 2007).

Consistent with a role in the transport of vDNA-NSP complex, NIG interacts *in vivo* and *in vitro* with different begomovirus NSPs, promotes the translocation of NSP from the nucleus to the cytosol and displays a proviral function (Carvalho et al., 2008a). Furthermore, NIG shares structural features and transport proprieties with hRIP, an essential Rev cofactor that accessorizes the release of HIV-1 RNAs from nuclear- exported complexes into the cytosol (Sánchez-Velar et al., 2004). Therefore, the begomovirus NSP role in plants may be similar to the HIV Rev function in mammals. Both hRIP and NIG exhibit an N-terminal ArfGAP domain, involved in in addressing intracellular protein localization, vesicular trafficking or signaling (Chavrier and Goud, 1999; Turner et al., 2001; Sabe et al., 2006; Bist et al., 2017). Based on its effects on NSP function and transport properties, NIG may function as a facilitator for the disassembly of nuclear-exported complexes at the cytoplasmic side, and/or a positive regulator for addressing these nuclear-exported proteins to specific sites within the cytoplasm (Carvalho et al., 2008a). Nevertheless, cytosolic targets for NIG have not been identified yet, and its proviral function has not been examined in reverse genetics studies, which preclude a conclusive understanding of the underlying mechanism for NIG action. Currently, the only identified NIG partner is a nuclear body-forming protein, AtWWP1 (*Arabidopsis thaliana* WW domain-containing protein 1), which accumulates during viral infection and sequesters NIG into nuclear bodies (Calil et al., 2018).

*Cabbage leaf curl virus* (CaLCuV) NSP has been demonstrated to interact with a nuclear acetyltransferase, designated nuclear shuttle protein interactor (AtNSI), which may control the ss-vDNA-NSP nuclear export through acetylation of CP and histones (McGarry et al., 2003; Carvalho and Lazarowitz, 2004). AtNSI assembles into highly active multimeric enzyme complexes, which acetylate plant proteins that may be involved in differentiation and/or plant defense (Carvalho et al., 2006). NSP binding to AtNSI prevents its assembly into the highly active enzyme complexes, and hence NSP recruits AtNSI into newly synthesized ss-vDNA to acetylate bound CP. CP acetylation disrupts vDNA-CP interaction to favor vDNA-NSP complex formation and subsequent vDNA trafficking to the cytosol (Carvalho and Lazarowitz, 2004; Carvalho et al., 2006). AtNSI also acetylates histone H3, which is associated with vDNA nucleosome formation and genome assembly into minichromosomes, modulating viral replication and transcription (McGarry et al., 2003). Nevertheless, H3 also interacts with both NSP and MP from BDMV and has been shown to be part of the vDNA-protein complex that traffics intra- and intercellularly (Zhou et al., 2011). The presence of H3 into an NSP- and MP-containing movement-competent complex might be attributed to the histone packaging ability, thereby assisting the viral genome transport through the nucleoporin complex and subsequentially through plasmodesma. However, attempts to use gene silencing for assessing a direct H3 role in begomovirus infection have not been succeeded, and hence whether the putative proviral function of H3 is essential for infection remains to be determined. Nonetheless, these NSP-interacting host proteins with proviral functions may be in somehow involved in stabilizing the ss-vDNA-NSP complex or actively assisting the nucleocytoplasmic transport function of the viral protein.

Although the mechanism of intra- and intercellular transport of begomoviral DNA may exhibit species specificity in some aspects, a general model for the intracellular trafficking of vDNA holds that, in the nucleus, NSP binds to vDNA and H3, and the complex NSP-H3-vDNA is exported to the cytoplasm via nuclear pores. At the cytosolic side, the release of the trimeric complex is facilitated by NIG; then, NSP binds to MP that also binds to H3. The complex MP-NSP-H3-vDNA traffics to the cell periphery to be translocated to adjacent, uninfected cells via plasmodesmata. In these neighboring cells, NSP shuttles the viral genome to the nucleus to initiate new rounds of viral genome replication via the rolling circle mechanism (Krichevsky et al., 2006; Hanley-Bowdoin et al., 2013). Consistent with the general aspects of the intracellular movement of vDNA, the MP and NSP from AbMV and BDMV have been shown to confer cell-to-cell movement to recombinant mastreviral replicons, which belong to the *Mastrevirus* genus of the *Geminiviridae* family (Diamos et al., 2019). This lack of species specificity of the begomoviral MP and NSP functions depends on the replicon size and a fine-tuning expression of MP and NSP to prevent hypersensitive response and interference with replication and expression from viral replicons.

## NSP as a Suppressor of Plant Defenses

A host defense-suppressing function of NSP was first demonstrated through its interaction with the NSP-interacting kinases (NIKs), which belong to the superfamily of the transmembrane leucine-rich repeat receptor-like kinases (LRR-RLK) (Fontes et al., 2004; Mariano et al., 2004). The NSP-binding site on NIK1 from Arabidopsis corresponds to an 80-amino acid sequence delimited by amino acids 422–502, which overlaps with the putative active site for Ser/Thr kinases (subdomain VIb–HrDvKssNxLLD) and the activation loop (subdomain VII–DFGAk/rx, plus subdomain VIII–GtxGyiaPEY). The activation site of NIK1 corresponds to a threonine residue at position 474 within the activation loop of the kinase (Fontes et al., 2004; Santos et al., 2009). As a single-pass transmembrane receptor kinase, NIK1 may dimerize or multimerize with itself and/or receptors to promote transphosphorylation and subsequent kinase activation (Santos et al., 2010). Binding of NSP to NIK1 blocks the phosphorylation of the crucial threonine-474 residue and hence prevents the activation of NIK1 that otherwise would transduce an antiviral signal in response to begomovirus infection. The NSP-NIK complex formation is neither virus-specific nor host-specific (Fontes et al., 2004; Mariano et al., 2004). The binding of NSP to NIKs is conserved among NSP from different begomoviruses, such as CaLCuV, *Tomato golden mosaic virus* (TGMV), *Tomato crinkle yellow leaf virus* (TCrYLV), *Tomato yellow spot virus* (ToYSV), and NIKs from different plant species, including soybean, tomato and Arabidopsis (Fontes et al., 2004; Mariano et al., 2004; Sakamoto et al., 2012). This observation has led to the manipulation of the NIK1 immune receptor-like kinase as a target for engineering broad-range resistance to begomoviruses (Brustolini et al., 2015). As a proof of concept, the constitutive activation of NIK1 by expression of the NIK1-T474D gain-of-function mutant resulted in suppression of general translation and enhanced resistance to different species of tomato-infecting begomoviruses in tomato transgenic lines. Despite 25% suppression of translation, the transgenic lines were phenotypically indistinguishable from untransformed tomato plants under greenhouse optimized growth conditions. In contrast, the constitutive activation of the NIK1-mediated antiviral signaling in Arabidopsis caused stunted growth (Zorzatto et al., 2015). An additional pitfall in engineering NIK1-mediated broad-range resistance to begomoviruses in crops is the finding that NIK1 negatively modulates antibacterial immunity (Li et al., 2019). NIK1 overexpression in Arabidopsis has been shown to enhance susceptibility to bacterial infections, which is not desirable under field conditions where plants are often exposed to simultaneous infections by different pathogens (Li et al., 2019).

Progress in elucidating the NIK1-mediated signaling pathway includes the characterization of the downstream components, the ribosomal protein L10 (RPL10), and the transcription-repressing factor L10-interacting Myb domain-containing protein (LIMYB) (Carvalho et al., 2008c; Rocha et al., 2008; Zorzatto et al., 2015). More recently, the NIK1-mediated antiviral signaling has been demonstrated to be activated by begomovirus-derived nucleic acids that very likely function as pattern-associated molecular patterns (PAMPs) to induce or stabilize the oligomerization of NIK1 as the critical early event that triggers signaling and transduction from a receptor (Teixeira et al., 2019).

The mechanistic model for NIK1 activation and defense assembly holds that, in response to virus infection, begomovirus-derived nucleic acids function as PAMPs to mediate dimerization of NIK1 with itself, its paralog NIK2 or another unknown transmembrane receptor, which may function as a PAMP recognition receptor (PRR) (Teixeira et al., 2019). The NIK1 oligomerization induces the phosphorylation of the NIK1 kinase domain at the key threonine residue at position 474 (Machado et al., 2015, 2017). This phosphorylation-induced activation of NIK1 mediates the phosphorylation of the downstream component RPL10, which, in turn, is reallocated from the cytoplasm to the nucleus, where it binds to LIMYB. The RPL10-LYMIB complex represses the expression of translational machinery-related genes, including initiation and elongation factors of translation and ribosomal protein genes (Calil and Fontes, 2017; Gouveia et al., 2017). Prolonged downregulation of the translational machinery of plant cells leads to the suppression of global translation (Brustolini et al., 2015; Zorzatto et al., 2015). The viral mRNAs are not able to escape this translational regulatory mechanism of plant cells; they are not efficiently translated, compromising infection (Brustolini et al., 2015). In host-begomovirus compatible interactions, NSP from begomoviruses binds to the NIK1 kinase domain to prevent activation of the NIK1-mediated antiviral signaling creating an environment that is more favorable to virus infection (Fontes et al., 2004; Santos et al., 2009). Therefore, this antiviral signaling pathway is a defense strategy evolutionarily overcome by the virus.

Nuclear shuttle protein has also been shown to interact with two other receptor-like kinases, the brassinosteroid insensitive 1-associated kinase 1 (BAK1) and a PERK-like kinase, designated NSP-associated kinase (NsAK) (Florentino et al., 2006; Li et al., 2019). While the NSP-NsAK complex formation positively contributes to viral infection, the interaction of NSP with BAK1 may be associated with a host defense-suppressing function. BAK1 functions as a co-receptor of several PRRs, such as flagellin sensing 2 (FLS2) and elongation factor thermo unstable receptor (EFR), which perceive specific PAMPs and trigger or amplify PTI (PAMP-triggered immunity), the first layer of the plant innate immune system (Zipfel et al., 2006; Chinchilla et al., 2007). Several lines of evidence indicate that NSP may suppress BAK1 function. First, NIK1 and BAK1 are structurally related; they belong to the same subfamily II of LRR-RLKs and share conserved positions for the activation site of the kinases (Sakamoto et al., 2012). Second, the binding site of NSP on NIK1 and the corresponding sequence on BAK1 are highly conserved (Santos et al., 2010). Third, despite its antiviral function, NIK1 has also been shown to inhibit BAK1-mediated PTI through direct interaction with the co-receptor BAK1 and the PRR FLS2 (Li et al., 2019). The viral NSP suppressor of NIK1 may interfere with the NIK1-BAK1-FLS2 complex formation. Finally, viral infection has been shown to inhibit PTI directly via viral protein suppressors, including the coat protein (CP) from *Plum pox virus* (PPV; Nicaise and Candresse, 2017), the movement protein (MP) from *Cucumber mosaic virus* (CMV; Kong et al., 2018) and P6 from *Cauliflower mosaic virus* (CaMV) (Zvereva et al., 2016). PPV CP has also been shown to function as a viral effector (Avr factor), which activates effector-triggered immunity (ETI), the second layer of the innate immune system, via specific recognition by a host intracellular receptor, the resistance R protein (Decroocq et al., 2009). This finding suggests that viral suppressors may link the suppression of PTI with activation of ETI, as predicted by the zig-zag evolutionary model of plant innate immunity (Jones and Dangl, 2006). NSP from BDMV has also been shown to function as an Avr factor for ETI activation in resistant *Phaseolus vulgaris* genotypes (Garrido-Ramirez et al., 2000), linking a primary mechanism of antiviral immunity at the cell surface (NIK1 and BAK1) with ETI.

Nuclear shuttle protein from CaLCuV also interacts with ASYMMETRIC LEAVES2 (AS2), which regulates positively mRNA decapping and degradation, inhibits siRNA accumulation, and functions as an endogenous suppressor of post-transcriptional gene silencing (PTGS) (Ye et al., 2015). As a suppressor of host defense, NSP induces the expression of AS2 and also causes the nuclear exit of AS2 to activate DCP2 decapping activity and suppress PTGS. Therefore, NSP stimulates the AS2 proviral function and hence increases the susceptibility to begomovirus in Arabidopsis.

Immune-affinity capture of proteins using GFP-tagged-NSP (from AbMV) identified a unique peptide from the Arabidopsis Ras-GAP SH3 domain-binding protein (G3BP) homolog as a candidate for NSP-interacting protein (Krapp et al., 2017). G3BP is a stress granule (SG)-localized protein, which is required for SG assembly, implicated in host defense against animal viruses (Tourrière et al., 2003; Lloyd, 2012). The mRNA-protein aggregates of SGs are formed upon biotic and abiotic stresses in response to stalled translation (Buchan and Parker, 2009). Viral proteins bind G3BP via their FGDF motifs to impair SG assembly (McInerney, 2015; Panas et al., 2015). Additional evidence that AbMV NSP binds AtG3BP *in planta* relies on the observation that mutated NSP on its FGDF-like motif loses the capacity to co-localize with AtG3BP in SG, under stress conditions (Krapp et al., 2017). The biological relevance of the G3BP-NSP interaction has not been investigated; thereby, the proposition that NSP binds to G3BP to inhibit SG formation is reminiscent of functional studies of animal virus-host interactions (Lloyd, 2012; Panas et al., 2015).

## An NSP-Interacting Immune Hub Against Begomoviruses

**FIGURE 1:**
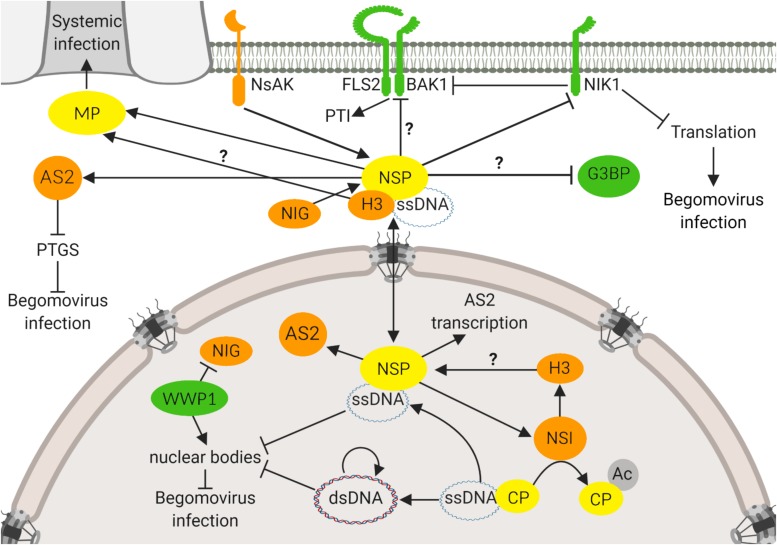
NSP-interacting immune hub. The NSP-interacting immune hub consists of host proteins displaying antiviral functions (green) and proviral functions (orange). Viral proteins are indicated in yellow. In the nucleus, CaLCuV NSP binds to AtNSI to facilitate the acetylation of CP that favors NSP-vDNA complex formation. BDMV NSP also binds to H3. CaLCuV NSP increases the expression of proviral AS2, an inhibitor of PTGS, and also promotes its reallocation to the cytosol. At the cytosolic, perinuclear region, CaLCuV NSP interacts with NIG to facilitate the release of the vDNA-NSP complex from the nuclear pore into the cytosol. To prevent the NIG proviral function, the antiviral protein WWP1 sequesters NIG into nuclear bodies (NB), impairing its cytosolic transport function. As a counter defensive measure, vDNA disrupts the WWP1-NB assembly. At the cytoplasm, NSP may interact with G3BP, a protein required for the assembly of stress granules. NSP also interacts with the viral MP, which may provide the directionality for the NSP-vDNA-H3 complex toward the plasmodesmata for translocation to the neighboring cells. At the plasma membrane, NSP interacts with the receptor-like kinase NIK1 to suppress its kinase activity that otherwise would transduce an antiviral signal against begomoviruses. NIK1 also inhibits PTI, via interaction with BAK1 that may also be a target of the suppressing activity of NSP. NSP may serve as a substrate for another receptor-like kinase, NsAK, at the plasma membrane. Question marks denote not fully characterized proviral or antiviral functions of host proteins. Arrows indicate a positive effect on protein activity, and inhibitor lines denote suppressing activities. Double arrow indicates the double direction of protein movement. The figure was created with BioRender.com.

Genome-wide studies of plant immunity and pathogen infection strategies have revealed an integrated picture of the plant-pathogen interactions, in which the pathogen effector interactions and plant defense proteins converge to subsets of highly interconnected host proteins, designated hubs (Mukhtar et al., 2011). Based on its intra- and intercellular transport function, NSP from begomoviruses may be an excellent target for identifying components of the basic cellular processes as this viral protein interacts with host factors in different organelles and may represent itself a hub for host protein-protein interactions (Figure 1). Accordingly, we summarized here that the NSP-interacting immune hub is formed with proteins located in different subcellular compartments, including the nuclear acetylase, AtNSI (Carvalho and Lazarowitz, 2004), the nuclear Histone H3 (Zhou et al., 2011), the cytosolic GTPase NIG (Carvalho et al., 2008a), the nucleocytoplasmic AS2 (Ye et al., 2015), the cytoplasmic G3BP (Krapp et al., 2017) and the plasma membrane receptor kinases, designated NIKs (Fontes et al., 2004; Mariano et al., 2004) and NsAKs (Florentino et al., 2006). This NSP-interacting immune hub accommodates proviral proteins, including AtNSI, H3, NIG, NsAK, and AS2 and antiviral proteins, such as NIKs, G3BP, and AtWWP1. The proviral factors may facilitate or stabilize NSP binding to vDNA, such as AtNSI and H3, or may direct an active intracellular transport of NSP-vDNA, like NIG. The NIG-NSP interaction seems to be very important during infection because plant cells have evolved a defense strategy to prevent the NIG proviral function (Calil et al., 2018). Viral infection promotes the accumulation of a nuclear body (NB)-forming protein, AtWWP1, which relocates NIG from the cytoplasm to the nucleus where it is confined to AtWWP1-NBs, impairing the NIG cytosolic transport function (Calil et al., 2018). The antiviral function of AtWWP1-NBs, however, may be antagonized by the viral infection. As a counter defensive measure, vDNA binds to AtWWP1 and induces either a decrease in the number or disruption of AtWWP1-NBs, restoring the NIG cytosolic localization (Calil et al., 2018). Therefore, the begomoviruses have evolved vDNA-based virulence strategies to overcome nuclear bodies-derived host defense responses.

Likewise, the binding of NSP to defense proteins, such as NIK1 and AS2, leads to suppression of host immunity. In the first case, during infection, NSP inhibits the NIK1 kinase that otherwise would transduce an antiviral signal to protect plants against begomoviruses (Brustolini et al., 2015; Machado et al., 2015, 2017). In the latter case, NSP not only increases AS2 expression, an endogenous suppressor of PTGS, but also can induce nuclear export of AS2 to the cytosol where it interacts with DCP2 to stimulate decapping activity, decrease siRNA accumulation and impair RNA silencing (Ye et al., 2015).

The NSP-interacting immune hub also cross-talks with the antibacterial immune response through interactions between NIK1 and the flagellin receptor FLS2 and its co-receptor BAK1 (Li et al., 2019). In the absence of bacterial and viral infection, NIK1 binds to FLS2 and BAK1 to prevent the activation of an autoimmune response. NIK1 may control FLS2-BAK1 complex formation, as the amplitude of the immune response depends on basal concentrations of NIK1 (Li et al., 2019). The bacterial infection leads to the formation of an active immune complex, between PRR FLS2 and its co-receptor BAK1, which phosphorylates bound NIK1 at the crucial Thr-474 to activate antiviral immunity. Therefore, bacterial infection may promote plant resistance to begomoviruses in a NIK1-dependent manner.

The NSP-interacting immune hub was integrated into the Arabidopsis interactome (BioGRID database)^1^ and (Arabidopsis interactome database)^2^, and the LRR-based cell surface interaction network (CSILRR; Smakowska-Luzan et al., 2018), using Cytoscape software^3^ (Supplementary Figure 1 and Supplementary Table 1). This procedure identified the NSP-host protein-protein interaction (PPI) network, containing directly and indirectly NSP-interacting host proteins that converge to relevant hubs of plant-pathogen interactions. Although this *in silico* studies revealed that the functional characterization of a potential NSP-interacting immune hub is far from being complete, these NSP-derived hubs may represent a framework for rationalizing future studies in begomovirus-host interactions.

## Conclusion

Successful systemic plant infection by viruses depends on spreading their genomes between cells and throughout the plant. Thus, the identification of host factors involved in virus movement that interact direct or indirectly with virus-encoded movement proteins is essential for the establishment of novel antiviral strategies. NSP may use the nuclear export machinery to facilitate the translocation of viral DNA from the nucleus to the cytoplasm. Although some progress in the identification of NSP partners has been made in the last decade, the characterization of a potential NSP-interacting immune hub is far from being complete. For instance, we now know that intracellular trafficking of NSP-DNA complexes is accessorized by NIG at the cytosolic side, but the transport activity and underlying mechanism for NIG function are poorly understood (Carvalho et al., 2008a, b). Evidence that NIG functions in the viral DNA-NSP trafficking relies on the demonstration that NIG interacts *in vivo* and *in vitro* with NSP, is capable of redirecting NSP-DNA complexes from the nucleus to the cytoplasm and functions as a proviral factor during begomovirus infection (Carvalho et al., 2008a, b). These findings suggest that NIG may be a susceptibility gene for engineering resistance to begomovirus. Nevertheless, a conclusive demonstration that NIG is essential for begomovirus infection is lacking, and cytosolic NIG-interacting proteins that could provide an intracellular route for the complex vDNA-NSP trafficking toward the plasmodesmata have not been identified.

The host defense-suppressing function of NSP has also been characterized via protein-protein interaction studies. In this regard, NSP negatively controls virus silencing by inducing the expression and activity of the endogenous inhibitor of PTGS, AS2 (Ye et al., 2015). NSP also negatively modulates innate immune responses by suppressing activity of the immune receptor-like kinases NIK1 and BAK1 (Fontes et al., 2004; Sakamoto et al., 2012). While compelling evidence demonstrates that NSP inhibits the phosphorylation-mediated activation of NIK1 to prevent the receptor from signaling (Calil and Fontes, 2017; Gouveia et al., 2017), evidence that NSP controls negatively PTI is still rudimentary. Although NSP has been shown to interact with BAK1, a co-receptor of PTI, the NSP-mediated suppression of BAK1 function has not been examined yet. Therefore, attempts to deploy the innate immune responses as targets for plant resistance to begomovirus are still in their infancy.

## Author Contributions

LM, GR, NR, NE, VL, CD, and EF designed the concept and organized the manuscript. JS performed bioinformatic analysis. LM, GR, and EF wrote the manuscript. LM, CD, and EF edited the manuscript.

## Conflict of Interest

The authors declare that the research was conducted in the absence of any commercial or financial relationships that could be construed as a potential conflict of interest.

## Footnotes

1 http://www.thebiogrid.org

2 http://interactome.dfci.harvard.edu/A_thaliana/

3 https://cytoscape.org/

## References

[B1] Bist P. Kim S. S. Pulloor N. K. McCaffrey K. Nair S. K. Liu Y. (2017). ArfGAP domain-containing protein 2 (ADAP2) integrates upstream and downstream modules of RIG-I signaling and facilitates type I interferon production. *Mol. Cell. Biol.* 37:e00537-36. 10.1128/MCB.00537-16 PMC5335504 27956705

[B2] Brustolini O. J. B. Machado J. P. B. Condori-Apfata J. A. Coco D. Deguchi M. Loriato V. A. P. (2015). Sustained NIK-mediated antiviral signalling confers broad-spectrum tolerance to begomoviruses in cultivated plants. *Plant Biotechnol. J.* 13 1300–1311. 10.1111/pbi.12349 25688422 PMC4857726

[B3] Buchan J. R. Parker R. (2009). Review eukaryotic stress granules: the ins and outs of translation. *Mol. Cell* 36 932–941. 10.1016/j.molcel.2009.11.020 20064460 PMC2813218

[B4] Calil I. P. Fontes E. P. B. (2017). Plant immunity against viruses: antiviral immune receptors in focus. *Ann. Bot.* 119 711–723. 10.1093/aob/mcw200 27780814 PMC5604577

[B5] Calil I. P. Quadros I. P. S. Araújo T. C. Duarte C. E. M. Gouveia-Mageste B. C. Silva J. C. F. (2018). A WW domain-containing protein forms immune nuclear bodies against begomoviruses. *Mol. Plant.* 11 1449–1465. 10.1016/j.molp.2018.09.009 30296599

[B6] Carvalho C. M. Fontenelle M. R. Florentino L. H. Santos A. A. Zerbini F. M. Fontes E. P. B. (2008a). A novel nucleocytoplasmic traffic GTPase identified as a functional target of the bipartite geminivirus nuclear shuttle protein. *Plant J.* 55 869–880. 10.1111/j.1365-313X.2008.03556.x 18489709

[B7] Carvalho C. M. Machado J. P. B. Zerbini F. M. Fontes E. P. B. (2008b). NSP-interacting GTPase: a cytosolic protein as cofactor for nuclear shuttle proteins. *Plant Signal. Behav.* 3 752–754. 10.4161/psb.3.9.6641 19704847 PMC2634578

[B8] Carvalho C. M. Santos A. A. Pires S. R. Rocha C. S. Saraiva D. I. Machado J. P. B. (2008c). Regulated nuclear trafficking of rpL10A mediated by NIK1 represents a defense strategy of plant cells against virus. *PLoS Pathog.* 4:e1000247. 10.1371/journal.ppat.1000247 19112492 PMC2597721

[B9] Carvalho M. F. Lazarowitz S. G. (2004). Interaction of the movement protein NSP and the *Arabidopsis* acetyltransferase AtNSI is necessary infection and pathogenicity. *J. Virol.* 78 11161–11171. 10.1128/JVI.78.20.11161 15452236 PMC521842

[B10] Carvalho M. F. Turgeon R. Lazarowitz S. G. (2006). The geminivirus nuclear shuttle protein NSP inhibits the activity of AtNSI, a vascular-expressed *Arabidopsis* acetyltransferase regulated with the sink-to-source transition. *Plant Physiol.* 140 1317–1330. 10.1104/pp.105.075556 16461385 PMC1435821

[B11] Chavrier P. Goud B. (1999). The role of ARF and Rab GTPases in membrane transport. *Curr. Opin. Cell Biol.* 11 466–475. 10.1016/S0955-0674(99)80067-2 10449335

[B12] Chinchilla D. Zipfel C. Robatzek S. Kemmerling B. Nürnberger T. Jones J. D. G. (2007). A flagellin-induced complex of the receptor FLS2 and BAK1 initiates plant defence. *Nature* 448 497–500. 10.1038/nature05999 17625569

[B13] Decroocq V. Salvador B. Sicard O. Glasa M. Cosson P. Svanella-Dumas L. (2009). The determinant of potyvirus ability to overcome the RTM resistance of *Arabidopsis thaliana* maps to the N-terminal region of the coat protein. *Mol. Plant Microb. Interact.* 22 1302–1311. 10.1094/mpmi-22-10-1302 19737103

[B14] Diamos A. G. Crawford J. M. Mason H. S. (2019). Fine-tuning expression of begomoviral movement and nuclear shuttle proteins confers cell-to-cell movement to mastreviral replicons in *Nicotiana benthamiana* leaves. *J. Gen. Virol.* 100 1038–1051. 10.1099/jgv.0.001275 31107197

[B15] Florentino L. H. Santos A. A. Fontenelle M. R. Pinheiro G. L. Zerbini F. M. Baracat-Pereira M. C. (2006). A PERK-like receptor kinase interacts with the geminivirus nuclear shuttle protein and potentiates viral infection. *J. Virol.* 80 6648–6656. 10.1128/jvi.00173-06 16775352 PMC1488943

[B16] Fontes E. P. B. Santos A. A. Luz D. F. Waclawovsky A. J. Chory J. (2004). The geminivirus nuclear shuttle protein is a virulence factor that suppresses transmembrane receptor kinase activity. *Genes Dev.* 18 2545–2556. 10.1101/gad.1245904 15489295 PMC529541

[B17] Frischmuth S. Wege C. Hülser D. Jeske H. (2007). The movement protein BC1 promotes redirection of the nuclear shuttle protein BV1 of *Abutilon mosaic* geminivirus to the plasma membrane in fission yeast. *Protoplasma* 230 117–123. 10.1007/s00709-006-0223-x 17351736

[B18] Gafni Y. Epel B. L. (2002). The role of host and viral proteins in intra- and inter-cellular trafficking of geminiviruses. *Physiol. Mol. Plant Pathol.* 60 231–241. 10.1006/pmpp.2002.0402

[B19] Garrido-Ramirez E. R. Sudarshana M. R. Lucas W. J. Gilbertson R. L. (2000). Bean dwarf mosaic virus BV1 protein is a determinant of the hypersensitive response and avirulence in *Phaseolus vulgaris*. *Mol. Plant Microb. Interact.* 13 1184–1194. 10.1094/MPMI.2000.13.11.1184 11059485

[B20] Gouveia B. C. Calil I. P. Machado J. P. B. Santos A. A. Fontes E. P. B. (2017). Immune receptors and co-receptors in antiviral innate immunity in plants. *Front. Microbiol.* 7:2139 10.3389/fmicb.2016.02139 PMC5214455 28105028

[B21] Hanley-Bowdoin L. Bejarano E. R. Robertson D. Mansoor S. (2013). Geminiviruses: masters at redirecting and reprogramming plant processes. *Nat. Rev. Microbiol.* 11 777–788. 10.1038/nrmicro3117 24100361

[B22] Hehnle S. Wege C. Jeske H. (2004). Interaction of DNA with the movement proteins of geminiviruses revisited. *J. Virol.* 78 7698–7706. 10.1128/jvi.78.14.7698-7706.2004 15220444 PMC434128

[B23] Jones J. D. G. Dangl J. L. (2006). The plant immune system. *Nature* 444 323–329. 10.1038/nature05286 17108957

[B24] Kleinow T. Tanwir F. Kocher C. Krenz B. Wege C. Jeske H. (2009). Expression dynamics and ultrastructural localization of epitope-tagged *Abutilon mosaic* virus nuclear shuttle and movement proteins in *Nicotiana benthamiana* cells. *Virology* 391 212–220. 10.1016/j.virol.2009.06.042 19628237

[B25] Kong J. Wei M. Li G. Lei R. Qiu Y. Wang C. (2018). The cucumber mosaic virus movement protein suppresses PAMP-triggered immune responses in *Arabidopsis* and tobacco. *Biochem. Biophys. Res. Comm.* 498 395–401. 10.1016/j.bbrc.2018.01.072 29407169

[B26] Krapp S. Greiner E. Amin B. Sonnewald U. Krenz B. (2017). The stress granule component G3BP is a novel interaction partner for the nuclear shuttle proteins of the nanovirus pea necrotic yellow dwarf virus and geminivirus *Abutilon mosaic* virus. *Virus Res.* 227 6–14. 10.1016/j.virusres.2016.09.021 27693920

[B27] Krichevsky A. Kozlovsky S. V. Gafni Y. Citovsky V. (2006). Nuclear import and export of plant virus proteins and genomes. *Mol. Plant Pathol.* 7 131–146. 10.1111/j.1364-3703.2006.00321.x 20507434

[B28] Kumar R. V. (2019). Plant antiviral immunity against geminiviruses and viral counter-defense for survival. *Front. Microbiol.* 10:1460 10.3389/fmicb.2019.01460 PMC6607972 31297106

[B29] Lazarowitz S. G. Beachy R. N. (1999). Viral movement proteins as probes for intracellular and intercellular trafficking in plants. *Plant Cell* 11 535–548. 10.1105/tpc.11.4.535 10213776 PMC144200

[B30] Li B. Ferreira M. A. Huang M. Camargos L. F. Yu X. Teixeira R. M. (2019). The receptor-like kinase NIK1 targets FLS2/BAK1 immune complex and inversely modulates antiviral and antibacterial immunity. *Nat. Commun.* 10:4996. 10.1038/s41467-019-12847-6 31676803 PMC6825196

[B31] Li F. Yang X. Bisaro D. M. Zhou X. (2018). The βC1 protein of geminivirus-betasatellite complexes: a target and repressor of host defenses. *Mol. Plant* 11 1424–1426. 10.1016/j.molp.2018.10.007 30404041

[B32] Lloyd R. E. (2012). How do viruses interact with stress-associated RNA granules? *PLoS Pathog.* 8:e1002741. 10.1371/journal.ppat.1002741 22761570 PMC3386173

[B33] Machado J. P. B. Brustolini O. J. B. Mendes G. C. Santos A. A. Fontes E. P. B. (2015). NIK1, a host factor specialized in antiviral defense or a novel general regulator of plant immunity? *Bioessays* 37 1236–1242. 10.1002/bies.201500066 26335701

[B34] Machado J. P. B. Calil I. P. Santos A. A. Fontes E. P. B. (2017). Translational control in plant antiviral immunity. *Genet. Mol. Biol.* 40 292–304. 10.1590/1678-4685-gmb-2016-0092 28199446 PMC5452134

[B35] Mariano A. C. Andrade M. O. Santos A. A. Carolino S. M. B. Oliveira M. L. Baracat-Pereira M. C. (2004). Identification of a novel receptor-like protein kinase that interacts with a geminivirus nuclear shuttle protein. *Virology* 318 24–31. 10.1016/j.virol.2003.09.038 14972531

[B36] McGarry R. C. Barron Y. D. Carvalho M. F. Hill J. E. Gold D. Cheung E. (2003). A novel *Arabidopsis* acetyltransferase interacts with the geminivirus movement protein NSP. *Plant Cell* 15 1605–1618. 10.1105/tpc.012120 12837950 PMC165404

[B37] McInerney G. M. (2015). FGDF motif regulation of stress granule formation. *DNA Cell Biol.* 34 1–4. 10.1089/dna.2015.295.7 26101899

[B38] Mukhtar M. S. Carvunis A. R. Dreze M. Epple P. Steinbrenner J. Moore J. (2011). Independently evolved virulence effectors converge onto hubs in a plant immune system network. *Science* 333 596–601. 10.1126/science.1203659 21798943 PMC3170753

[B39] Nicaise V. Candresse T. (2017). Plum pox virus capsid protein suppresses plant pathogen-associated molecular pattern (PAMP)-triggered immunity. *Mol. Plant Pathol.* 18 878–886. 10.1111/mpp.12447 27301551 PMC6638313

[B40] Noueiry A. O. Lucas W. J. Gilbertson R. L. (1994). Two proteins of a plant DNA virus coordinate nuclear and plasmodesmal transport. *Cell* 76 925–932. 10.1016/0092-8674(94)90366-2 8124726

[B41] Panas M. D. Schulte T. Thaa B. Sandalova T. Kedersha N. Achour A. (2015). Viral and cellular proteins containing FGDF motifs bind G3BP to block stress granule formation. *PLoS Pathog.* 11:e004659. 10.1371/journal.ppat.1004659 25658430 PMC4450067

[B42] Pascal E. Sanderfoot A. A. Ward B. M. Medville R. Turgeon R. Lazarowitz S. G. (1994). The geminivirus BR1 movement protein binds single-stranded DNA and localizes the cell nucleus. *Plant Cell* 6 995–1006. 10.1105/tpc.6.7.995 8069108 PMC160495

[B43] Rocha C. S. Santos A. A. Machado J. P. B. Fontes E. P. B. (2008). The ribosomal protein L10/QM-like protein is a component of the NIK-mediated antiviral signaling. *Virology* 380 165–169. 10.1016/j.virol.2008.08.005 18789471

[B44] Rojas M. R. Macedo M. A. Maliano M. R. Soto-Aguilar M. Souza J. O. Briddon R. W. (2018). World management of geminiviruses. *Annu. Rev. Phytopathol.* 56 637–677. 10.1146/annurev-phyto-080615-100327 30149794

[B45] Rojas M. R. Noueiry A. O. Lucas W. J. Gilbertson R. L. (1998). Bean dwarf mosaic geminivirus movement proteins recognize DNA in a form- and size-specific manner. *Cell* 95 105–113. 10.1016/S0092-8674(00)81786-9 9778251

[B46] Sabe H. Onodera Y. Mazaki Y. Hashimoto S. (2006). ArfGAP family proteins in cell adhesion, migration and tumor invasion. *Curr. Opin. Cell Biol.* 18 558–564. 10.1016/j.ceb.2006.08.002 16904307

[B47] Sakamoto T. Deguchi M. Brustolini O. J. B. Santos A. A. Silva F. F. Fontes E. P. B. (2012). The tomato RLK superfamily: phylogeny and functional predictions about the role of the LRRII-RLK subfamily in antiviral defense. *BMC Plant Biol.* 12:229. 10.1186/1471-2229-12-229 23198823 PMC3552996

[B48] Sánchez-Velar N. Udofia E. B. Yu Z. Zapp M. L. (2004). hRIP, a cellular cofactor for rev function, promotes release of HIV RNAs from the perinuclear region. *Genes Dev.* 18 23–34. 10.1101/gad.1149704 14701878 PMC314270

[B49] Sanderfoot A. A. Ingham D. J. Lazarowitz S. G. (1996). A viral movement protein as a nuclear shuttle. The geminivirus *BR1* movement protein contains domains essential for interaction with BL1 and nuclear localization. *Plant Physiol.* 110 23–33. 10.1104/pp.110.1.23 8587985 PMC157690

[B50] Sanderfoot A. A. Lazarowitz S. G. (1995). Cooperation in viral movement: the geminivirus BL1 movement protein interacts with BR1 and redirects it from the nucleus to the cell periphery. *Plant Cell* 7 1185–1194. 10.1105/tpc.7.8.1185 12242403 PMC160943

[B51] Sanderfoot A. A. Lazarowitz S. G. (1996). Getting it together in plant virus movement: cooperative interactions between bipartite geminivirus movement proteins. *Trends Cell Biol.* 6 353–358. 10.1016/0962-8924(96)10031-3 15157433

[B52] Santos A. A. Carvalho C. M. Florentino L. H. Ramos H. J. O. Fontes E. P. B. (2009). Conserved threonine residues within the A-loop of the receptor NIK differentially regulate the kinase function required for antiviral signaling. *PLoS One* 4:e5781. 10.1371/journal.pone.0005781 19492062 PMC2686266

[B53] Santos A. A. Lopes K. V. G. Apfata J. A. C. Fontes E. P. B. (2010). NSP-interacting kinase, NIK: a transducer of plant defence signalling. *J. Exp. Bot.* 61 3839–3845. 10.1093/jxb/erq219 20624762

[B54] Smakowska-Luzan E. Mott G. A. Parys K. Stegmann M. Howton T. C. Layeghifard M. (2018). An extracellular network of *Arabidopsis leucine*-rich repeat receptor kinases. *Nature* 553 342–346. 10.1038/nature25184 29320478 PMC6485605

[B55] Teixeira R. M. Ferreira M. A. Raimundo G. A. S. Loriato V. A. P. Reis P. A. B. Fontes E. P. B. (2019). Virus perception at the cell surface: revisiting the roles of receptor-like kinases as viral pattern recognition receptors. *Mol. Plant Pathol.* 20 1196–1202. 10.1111/mpp.12816 31094066 PMC6715618

[B56] Tourrière H. Chebli K. Zekri L. Courselaud B. Blanchard J. M. Bertrand E. (2003). The RasGAP-associated endoribonuclease G3BP assembles stress granules. *J. Cell Biol.* 160 823–831. 10.1083/jcb.200212128 12642610 PMC2173781

[B57] Turner C. E. West K. A. Brown M. C. (2001). Paxillin-ARF GAP signaling and the cytoskeleton. *Curr. Opin. Cell Biol.* 13 593–599. 10.1016/S0955-0674(00)00256-8 11544028

[B58] Ye J. Yang J. Sun Y. Zhao P. Gao S. Jung C. (2015). Geminivirus activates ASYMMETRIC LEAVES 2 to accelerate cytoplasmic DCP2-mediated mRNA turnover and weakens RNA silencing in *Arabidopsis*. *PLoS Pathog.* 11:e1005196. 10.1371/journal.ppat.1005196 26431425 PMC4592220

[B59] Zerbini F. M. Briddon R. W. Idris A. Martin D. P. Moriones E. Navas-Castillo J. (2017). ICTV virus taxonomy profile: geminiviridae. *J. Gen. Virol.* 98 131–133. 10.1099/jgv.0.000738 28284245 PMC5802298

[B60] Zhang S. C. Wege C. Jeske H. (2001). Movement proteins (BC1 andBV1) of *Abutilon mosaic* geminivirus are cotransported in and between cells of sink but not of source leaves as detected by green fluorescent protein tagging. *Virology* 290 249–260. 10.1006/viro.2001.1185 11883189

[B61] Zhou Y. Rojas M. R. Park M.-R. Seo Y.-S. Lucas W. J. Gilbertson R. L. (2011). Histone H3 interacts and colocalizes with the nuclear shuttle protein and the movement protein of a geminivirus. *J. Virol.* 85 11821–11832. 10.1128/jvi.00082-11 21900168 PMC3209288

[B62] Zipfel C. Kunze G. Chinchilla D. Caniard A. Jones J. D. G. Boller T. (2006). Perception of the bacterial PAMP EF-Tu by the receptor EFR restricts Agrobacterium-mediated transformation. *Cell* 125 749–760. 10.1016/j.cell.2006.03.037 16713565

[B63] Zorzatto C. Machado J. P. B. Lopes K. V. G. Nascimento K. J. T. Pereira W. A. Brustolini O. J. B. (2015). NIK1-mediated translation suppression functions as a plant antiviral immunity mechanism. *Nature* 520 679–682. 10.1038/nature14171 25707794 PMC4779052

[B64] Zvereva A. S. Golyaev V. Turco S. Gubaeva E. G. Rajeswaran R. Schepetilnikov M. V. (2016). Viral protein suppresses oxidative burst and salicylic acid-dependent-autophagy and facilitates bacterial growth on virus-infected plants. *New Phytol.* 211 1020–1034. 10.1111/nph.13967 27120694

