# Supplementary material for: A Begomovirus Nuclear Shuttle Protein-Interacting Immune Hub: Hijacking Host Transport Activities and Suppressing Incompatible Functions
Source: Front Plant Sci. 2020 Apr 8;11:398. doi: 10.3389/fpls.2020.00398 (PMC7156597; doi:10.3389/fpls.2020.00398)
Supplement: Supplementary file 1 [file Data_Sheet_1.PDF]

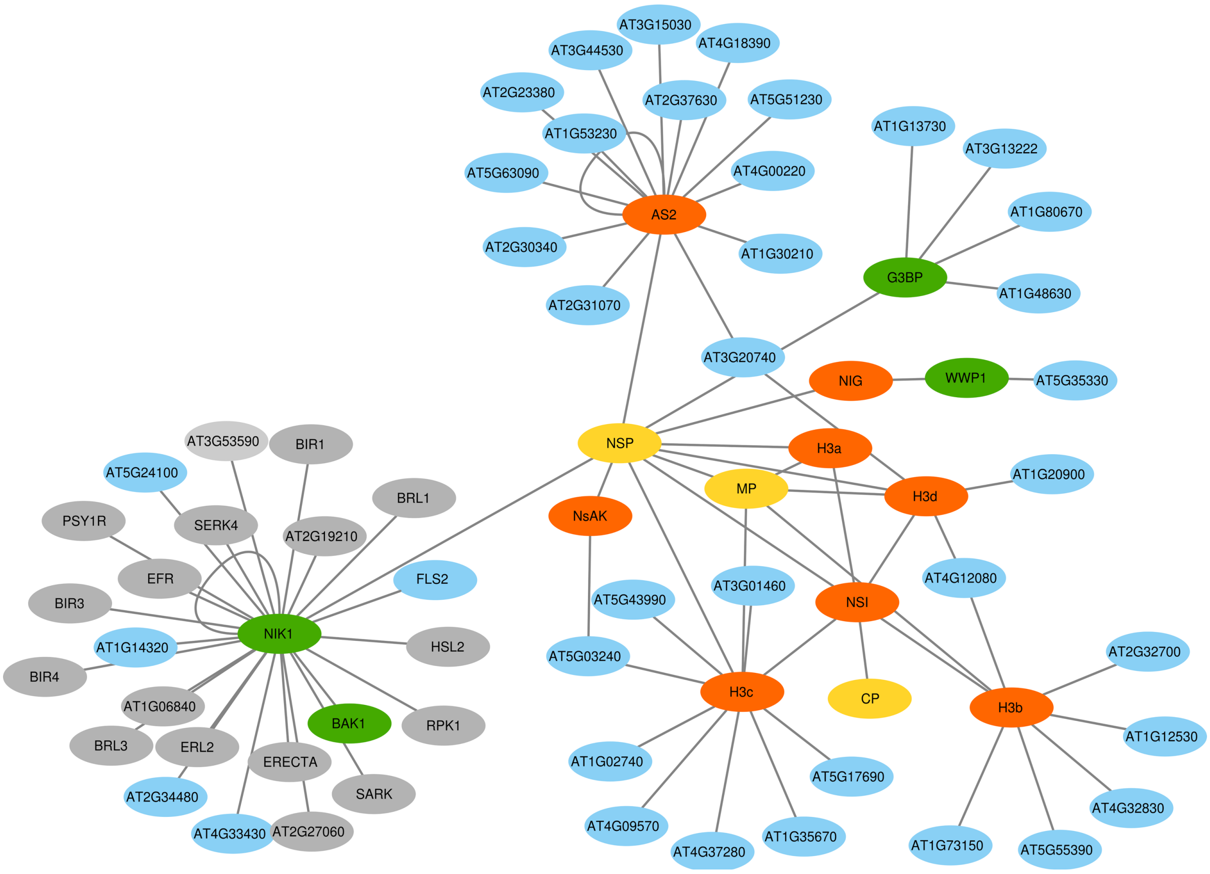

Supplementary Figure 1. NSP-Arabidopsis protein-protein interaction network. The NSP-interacting proteins were integrated into the Arabidopsis interactome and the LRR-based cell surface interaction network (CSILRR). The network displays a firework topology, which was assembled by the Cytoscape software. The viral proteins, NSP, MP and CP, are represented in yellow. NSP-interacting proteins displaying an antiviral function are depicted in green and with proviral function in orange. Blue proteins were derived from the Arabidopsis interactome and gray proteins, from CSILRR.
